# Injuries to the Stomatognathic System in Brazilian Jiu-Jitsu Athletes

**DOI:** 10.1038/s41598-019-44598-1

**Published:** 2019-06-03

**Authors:** R. A. Macêdo-Filho, T. R. Leal, A. M. R. Cardoso, D. J. S. Sarmento, F. D. Verli, S. A. Marinho

**Affiliations:** 1 Dentistry Course, State University of Paraiba (Universidade Estadual da Paraíba- UEPB), Campus VIII, R. Coronel Pedro Targino, s/n, Postal Code: 58.233-000 Araruna, PB Brazil; 2 Pathology Laboratory, Federal University of Jequitinhonha and Mucuri Valleys (Universidade Federal dos Vales do Jequitinhonha e Mucuri- UFVJM), Rua da Glória, 187, Building 02, Room 22, Postal Code: 39100-000 Diamantina, MG Brazil

**Keywords:** Dentistry, Epidemiology

## Abstract

As a contact sport, Brazilian jiu-jitsu requires the fighter to expose his/her stomatognathic system to the adversary, making him/her more susceptible to oral and maxillofacial injuries and disorders. The aim of the present study was to determine the prevalence of injuries and disorders of the stomatognathic system and associated factors among practitioners of Brazilian jiu-jitsu. A total of 179 athletes were interviewed and submitted to a physical examination. The majority was male, in the beginner category and had participated in competitions. Athletes with more experience had a higher frequency of orofacial injuries (PR = 1.77; 95% CI: 1.01–1.38), such as oral mucous lacerations and skin abrasions in the facial region, which mainly occurred during training sessions. A mouthguard is not mandatory for the sport and many athletes (both beginners and more experienced athletes) do not use one due to difficulty breathing with the device. A prefabricated (type II) mouthguard was the most common among the athletes who used this equipment, although it does not offer adequate protection. Athletes on more advanced levels wore mouthguards significantly more often (PR = 1.96; 95% CI: 1.11–2.45). In conclusion, more experienced jiu-jitsu athletes had a higher frequency of orofacial injuries, such as lacerations and abrasions, and are more likely to wear a mouthguard. However, longitudinal studies are needed in order to assess the possible causes and risks.

## Introduction

Athletic performance depends on physical preparation as well as an adequate balance among physical, psychological and biological aspects. The oral cavity is susceptible to injury during contact sports, which can compromise athletic performance. Athletes of all ages, styles and abilities are at risk of suffering injuries during sport activities^1^.

Brazilian jiu-jitsu is a wrestling modality practised by approximately 550,000 Brazilian athletes. As a contact sport that requires the exposure of the entire stomatognathic system to an adversary, practitioners of jiu-jitsu are susceptible to oral-maxillofacial injuries and disorders during both training sessions and competitions^2–5^. Besides soft tissue damage, traumatic dental injury is prevalent during the practice of the sport. However, this type of trauma could be prevented with the use of a mouthguard^1,6^. The purpose of this oral appliance is to protect the teeth, soft tissues and other intraoral structures by dampening and distributing the forces of impact, with the upper portion protecting the soft tissues and anterior teeth and the lower portion avoiding mandibular contusions or fractures as well as trauma to the temporomandibular joints^7^. Indeed, increase in competitiveness and the number of practitioners of contact sports have led to an increase in the occurrence of trauma stemming from fighting and physical contact during competitions^8^.

The aims of the present study were to determine the prevalence of injuries and disorders of the stomatognathic system and associated factors in practitioners of Brazilian jiu-jitsu and investigate the use of mouthguards.

## Results

The data are displayed in Tables 1–4.

**Table 1:** Possession of mouthguard by Brazilian jiu-jitsu athletes.

Variables	Mouthguard		Bivariate		Multivariate	
	No	Yes	Unadjusted PR*		Adjusted PR†	
	n (%)	n (%)	p-value	(95% CI)	p-value	(95% CI)
**Sex**						
Male	97 (60.6)	63 (39.4)	0.169	1.870 (0.767–4.562)	—	—
Female	15 (78.9)	4 (21.1)		1.00	—	—
**Age group**						
16 to 24 years	70 (64.8)	38 (35.2)	0.412	0.704 (0.304–1.630)	—	—
25 to 39 years	39 (60.0)	26 (40.0)	0.608	0.800 (0.341–1.879)	—	—
More than 40 years	3 (50.0)	3 (50.0)		1.00	—	—
**Athletic level**						
Advanced	14 (42.4)	19 (57.6)	<0.001	1.977 (1.299–3.009)	0.020	1.962 (1.114–2.456)
Intermediary	25 (58.1)	18 (41.9)	0.125	1.437 (0.904–2.285)	0.673	1.115 (0.672–1.852)
Beginner	73 (70.9)	30 (29.1)		1.00	—	1.00
**Participation in competitions**						
Yes	76 (57.6)	56 (42.4)	0.035	1.813 (1.042–3.155)	—	—
No	36 (76.6)	11 (23.4)		1.00	—	—
**Time of practice**						
6–60 months	98 (65.8)	51 (34.2)	0.011	0.513 (0.307–0.857)	—	—
61–132 months	11 (52.4)	10 (47.6)	0.306	0.714 (0.375–1.360)	—	—
133–240 months	3 (33.3)	6 (66.7)		1.00	—	—
**Weekly practice**						
1–3 times	87 (64.9)	47 (35.1)	0.246	0.789 (0.529–1.177)	—	—
More than 3 times	25 (55.6)	20 (40.4)		1.00	—	—
**Duration of training**						
1–2 hours	109 (63.4)	63 (36.6)	0.194	0.641 (0.328–1.254)	—	—
More than 2 hours	3 (42.9)	4 (57.1)		1.00	—	—
**Injury**						
Absent	26 (74.3)	9 (25.7)	0.141	0.638 (0.351–1.160)	—	—
Present	86 (59.7)	58 (40.3)		1.00	—	—
**Tooth fracture**						
Yes	13 (39.4)	20 (60.6)	0.003	1.757 (1.204–2.557)	—	—
No	72 (65.5)	38 (34.5)		1.00	—	—
**Traumatic bone injury**						
Yes	17 (39.4)	20 (60.6)	0.392	1.210 (0.782–1.873)	—	—
No	72 (65.5)	38 (34.5)		1.00	—	—
**Laceration in oral mucosa**						
Yes	73 (57.9)	53 (42.1)	0.292	1.514 (0.699–3.279)	—	—
No	13 (72.2)	5 (27.8)		1.00	—	—
**Skin abrasion on face**						
Yes	54 (55.7)	44 (43.3)	0.202	1.359 (0.848–2.178)	—	—
No	31 (67.4)	15 (32.6)		1.00	—	—
**Limited mouth opening**						
Yes	3 (50.0)	3 (50.0)	0.615	1.236 (0.541–2.823)	—	—
No	81 (59.6)	55 (40.4)		1.00	—	—

*Poisson regression not adjusted for independent variables and possession of mouthguard.

**Variables incorporated into multivariate model (p < 0.20): gender, athletic level, participation in competition, time of practice, training duration, injuries and tooth fracture.

^†^Multivariate Poisson regression adjusted using backward stepwise procedure.

**Table 2:** Possession and use of mouthguard by Brazilian jiu-jitsu athletes.

Variables	Frequency n (%)
**Mouthguard**	
Yes	67 (37.4)
No	112 (62.6)
**Negligence of mouthguard possession (n = 112)**	
Lack of interest	50 (44.6)
Lack of Awareness	41 (36.6)
Cost	21 (18.8)
**Type of mouthguard (n = 67)**	
Type I	2 (3.0)
Type II	65 (97.0)
Type III	0 (0)
**Mouthguard use in training (n = 67)**	
Always	20 (29.8)
Never/Sometimes	47 (70.2)
Negligence of mouthguard use in training (n = 61)*	
Difficulty breathing	21 (34.4)
Discomfort	13 (21.3)
Loss of agility	9 (14.7)
Nausea	7 (11.4)
Traumatism	5 (8.1)
Neglect/omission	4 (6.8)
Increased salivation	2 (3.3)
**Mouthguard use in competitions (n = 67)**	
Always	39 (58.2)
Never/Sometimes	28 (41.8)
Negligence of mouthguard use in competitions (n = 38)*	
Difficulty breathing	16 (42.2)
Loss of agility	7 (18.4)
Discomfort	6 (15.7)
Nausea	5 (13.1)
Traumatism	2 (5.3)
Increased salivation	2 (5.3)
Neglect/omission	0 (0)

*Exhaustive variable, that allows participant to choose more than one option.

**Table 3:** Presence of orofacial injury in Brazilian jiu-jitsu athletes in bivariate and multivariate Poisson regression models.

Variables	Orofacial Injury		Bivariate		Multivariate	
	Present	Absent	Unadjusted PR*		Adjusted PR†	
	n (%)	n (%)	p-value	(95% CI)	p-value	(95% CI)
**Sex**						
Male	30 (18.8)	130 (81.2)	0.492	1.103 (0.834–1.457)	—	—
Female	5 (26.3)	14 (73.7)		1.00	—	—
**Age group**						
16 to 24 years	27 (25)	81 (75.0)	0.689	1.125 (0.632–2.002)	—	—
25 to 39 years	6 (9.2)	59 (90.8)	0.290	1.362 (0.769–2.410)	—	—
More than 40 years	2 (33.3)	4 (66.7)			—	—
**Athletic level**						
Advanced	2 (6.1)	31 (93.9)	<0.001	1.325 (1.140–1542)	0.047	1.777 (1.002–1.382)
Intermediate	3 (7)	40 (93.0)	<0.001	1.313 (1.131–1.523)	0.019	1.196 (1.030–1.389)
Beginner	30 (29.1)	73 (70.9)				1.00
**Participation in competition**						
Yes	17 (12.9)	115 (87.1)	<0.004	1.412 (1.117–1.785)	—	—
No	18 (38.3)	29 (61.7)		1.00	—	—
Time of practice						
6–60 months	33 (22.1)	116 (77.9)	0.292	0.876 (0.685–1.121)	—	—
61–132 months	1 (4.9)	20 (95.1)	0.589	1.071 (0.834–1.376)	—	—
133–240 months	1 (11.1)	8 (88.9)			—	—
**Weekly practice**						
1–3 times	27 (20.1)	107 (79.9)	0.720	0.971 (0.827–1.140)	—	—
More than 3 times	8 (17.8)	37 (82.2)		1.00	—	—
**Duration of training**						
1–2 hours	35 (20.3)	137 (79.7)	<0.001	0.797 (0.739–0.859)	—	—
More than 2 hours	0 (0)	7 (100.0)		1.00	—	—
**Possession of a mouthguard**						
Yes	9 (13.4)	58 (86.6)	0.090	1.127 (0.981–1.295)	—	—
No	26 (23.2)	86 (76.8)		1.00	—	—
**Dental arch coverage**						
Single	4 (15.4)	22 (84.6)	0.716	0.964 (0.789–1.177)	—	—
Double	5 (12.2)	36 (87.8)		1.00	—	—
**Use of mouthguard in training**						
Always	2 (10.0)	18 (90.0)	0.562	1.057 (0.876–1.277)	—	—
Never/Sometimes	7 (14.9)	40 (85.1)		1.00	—	—
**Use of mouthguard in competitions**						
Always	6 (15.4)	33 (84.6)	0.570	0.948 (0.787–1.141)	—	—
Never/Sometimes	3 (10.7)	25 (89.3)		1.00	—	—

*Poisson regression not adjusted for independent variables and presence of orofacial injury.

**Variables incorporated into multivariate model (p < 0.20): athletic level, participation in competition, duration of training, mouthguard use.

^†^Multivariate Poisson regression adjusted using backward stepwise procedure.

**Table 4:** Previous and current injuries in Brazilian jiu-jitsu athletes in bivariate and multivariate Poisson regression models.

Variables	Frequency	
	Previous injury n (%)	Current injury n (%)
**Injury**		
Yes	144 (80.4)	22 (12.4)
No	35 (19.6)	156 (87.6)
**Location**	**(n = 144)**	**(n = 22)**
Training	113 (78.5)	22 (100.0)
Competition	1 (0.7)	0 (0)
Both	30 (20.8)	0 (0)
Types of injury*	**(n = 294)**	**(n = 27)**
Laceration of oral mucosa	126 (42.6)	12 (44.5)
Skin abrasion on face	97 (32.9)	9 (33.3)
Tooth fracture	33 (11.2)	4 (14.4)
Traumatic bone injury	32 (10.8)	1 (3.7)
Limited mouth opening	6 (2.2)	1 (3.7)
Traumatic bone injury*	**(n = 32)**	**(n = 1)**
Nasal	17 (53.1)	1 (100.0)
Orbital	6 (18.8)	0 (0)
Zygomatic	3 (9.4)	0 (0)
Mandible	6 (18.8)	0 (0)
Type and symptoms of traumatic bone injury*	**(n = 32)**	**(n = 1)**
Contusion	12 (37.5)	1 (100.0)
Eyelid edema	6 (18.8)	0 (0)
Noose fracture	5 (15.6)	0 (0)
Contusion and chewing difficulty	5 (15.5)	0 (0)
Hematoma and zygomatic pain	3 (9.4)	0 (0)
Luxation of mandible	1 (3.1)	0 (0)
Mucosa laceration*	**(n = 217)**	**(n = 16)**
Lower Lip	107 (49.3)	10 (62.5)
Upper Lip	79 (36.4)	5 (31,3)
Oral Mucosa	31 (14.3)	1 (6,2)
Abrasion on facial skin*	**(n = 208)**	**(n = 14)**
Cheek	71 (34.2)	4 (28.4)
Orbital region	58 (27.8)	3 (21.5)
Noose	35 (16.9)	3 (21.5)
Frontal region	27 (12.9)	2 (14.3)
Mental region	17 (8.2)	2 (14.3)

*Exhaustive variable, that allows participant to choose more than one option.

The occurrence of centric bruxism and temporomandibular disorder (TMD) was non-significant in the sample. Only 4.5% (n = 8) of the participants could be presumptively diagnosed with bruxism and only 1.1% (n = 2) met the classification for TMD employed in this study. The Poisson regression analyses revealed that athletes on the advanced level had a greater chance of owning a mouthguard (PR = 1.96; 95% CI: 1.11 to 2.45) (Table 1) and those on the advanced (PR = 1.77; 95% CI: 1.01 to 1.38) and intermediate (PR = 1.19; 95% CI: 1.03 to 1.32) levels had a greater probability of having suffered an orofacial injury (Table 3).

## Discussion

Brazilian jiu-jitsu is a contact sport. This wrestling modality is widely practised in Brazil, with more than 550 thousand professional and amateur athletes in the country. However, the sport leaves athletes susceptible to injuries to the entire body, especially the stomatognathic system, which is the area of concern of dentists^1,9,10^. In the present study, 179 athletes of Brazilian jiu-jitsu were evaluated for injuries to the stomatognathic system stemming from the practice of the sport. A large proportion of the participants were younger than 24 years of age and the prevalence of orofacial injuries was high in these individuals^2,4,5,11^, occurring, mainly during training sessions.

Although most participants were in the “beginner” category, a large portion had practised the sport for at least six months and had participated in competitions. Athletes on more advanced levels had a greater chance of owning a mouthguard and the association between these two variables was significant. However, this same group also had a greater probability of having suffered orofacial injuries despite the fact that the majority always wore a mouthguard during competitions. This was already expected because they have been exposed for longer than the beginners. However, longitudinal studies are needed in order to assess the possible causes and risks. These findings are in agreement with data described by Shirani *et al*.^4^ and Levin and Zadik^12^, who report that experienced athletes are more affected by serious injuries than beginners.

Although athletes on more advanced levels were more affected by orofacial injuries in the present study, the injuries were not very serious. Most were lacerations of the oral mucosa and skin abrasions, which are injuries for which the use of a mouthguard would not have had an effect. However, previous studies have demonstrated that amateur athletes are more prone to orofacial injuries than professional athletes^13,14^. Kreiswirth *et al*.^15^ found that more experienced athletes (brown and black belts) were at greater risk of injuries to the entire body than less experienced athletes, although no statistically significant differences were found. According to Zeraruk *et al*.^16^, athletes aged 18 years or older are at greater risk of suffering injuries in comparison to younger athletes. The present findings are in agreement with this observation, as older athletes have more experience and have reached a higher technical level. Rainey^17^ found that professional athletes of mixed martial arts had significantly more injuries (threefold higher injury rate) than amateur athletes. The most commonly injured body region was the head/neck/face (38.2%) and the most common type of injury found was contusion (29.4%), which is similar to the present findings.

Some hypotheses could be raised in this study. The first is that, although more advanced athletes had mouthguards, they neglected to use these oral appliances due to difficulty breathing and discomfort, especially during training sessions. Another hypothesis is that no athletes had a personalised (type III) mouthguard, which is the most adequate for the prevention of oral trauma^9,18^, or that the most prevalent injuries were not related to the use of a mouthguard, as tooth fracture was only the third most frequent injury in the present investigation.

With regard to the prevalence of tooth fractures, the majority of the present sample (62.6%) did not possess a mouthguard. The justifications of the athletes for not having a mouthguard were a lack of interest, a lack of awareness regarding the existence of this oral appliance and the cost involved. The use of a mouthguard is considered the most effective protection against injuries to oral structures in different sport modalities^19^. The American Dental Association (ADA)^20^ recommends the use of this appliance for 29 sports. However, its use in Brazil is only mandatory for boxing^21^. Among the few athletes who had a mouthguard in the present study (37.4% of the sample), a large portion neglected to use it, especially during training sessions due to difficulty breathing and discomfort. Among those who wore a mouthguard, most used prefabricated thermoplastic protectors (type II) on a single arch due to the lower cost ($10 to $20), which is in agreement with data described by Gay-Escoda^22^. However, this type of mouthguard is not the most recommended, as it does not remain in position during impact and does not adequately provide the redistribution of impact forces^23^. The desired characteristics are found in a personalised type III mouthguard, which is produced with the assistance of a dentist based on the individual anatomy of each athlete^12,18^. Moreover, a personalised mouthguard offers greater comfort, protection against traumatic dental injuries, better breathing and better adaptation and causes less nausea^23,24^. Despite being the most indicated, the cost of this type of mouthguard ($122) is one of the main setbacks to acquiring one. This was evidenced in the present investigation, as no participants possessed such a mouthguard.

The head and neck injuries that most affected the athletes in the present study were soft tissue injuries (laceration of the oral mucosa and abrasion of facial skin) and tooth fractures. These prevalent injuries were reported during the patient history and observed during the physical examination. Such findings are in agreement with data described by Vidovic-Stesevic^24^, who conducted a study involving practitioners of karate, which is another contact sport.

Vidovic-Stesevic^24^ and Dursun *et al*.^25^ stressed the importance of healthcare professionals in the development of preventive programs, including raising awareness regarding the risks of traumatic orofacial injuries and protection methods in order to reduce the incidence of injuries among practitioners of martial arts. Although a mouthguard is not mandatory during the practice of contact sports in Brazil, its use is important to the prevention of more serious injuries. Thus, the intervention of dentists is necessary for the the prevention and treatment of different orofacial injuries caused by the practice of Brazilian jiu-jitsu and other contact sports.

In conclusion, the practitioners of Brazilian jiu-jitsu evaluated in the present study were predominantly young males on the beginner and intermediate levels of the sport. The majority participated in competitions. Athletes with more experience had a higher frequency of orofacial injuries, such as lacerations to the oral mucosa and abrasions to the facial skin, which mainly occurred during training sessions. Significant associations were found between the prevalence of orofacial injuries and a higher technical level as well as the use of a mouthguard and a higher technical level. Most athletes did not wear a mouthguard and those who had this equipment mainly used a prefabricated (type II) one, although this type does not offer adequate protection. The use of a mouthguard was largely neglected due to factors such as difficulty breathing, discomfort and the loss of agility among both beginners and more experienced athletes. Despite its need, the use of a mouthguard is not mandatory in the practice of jiu-jitsu in Brazil.

## Methods

A quantitative cross-sectional study was conducted with 179 athletes older than 16 years of age enrolled at six jiu-jitsu academies in the state of Paraíba, Brazil. This study received approval from the Human Research Ethics Committee of the State University of Paraíba, Brazil, under process number 46268415.1.0000.5187. All methods were performed in accordance with the guidelines and Brazilian regulations.

The main outcome measure of interest of this study was the history of orofacial injury due some trauma occurred during sports practice, reported by the athlete.

The sample consisted of all athletes of both genders older than 16 years of age, practicing the sport for at least six months with a training frequency of four times per month for at least one hour of daily practice. The athletes were classified using the grading system recommended by the International Brazilian Jiu-jitsu Federation (IBJJF). Athletes younger than 18 years required a statement of informed consent signed by a parent or guardian. Adults who agreed to participate of this research also signed the informed consent. Among a total of 206 athletes enrolled in the academies, 179 (86.9%) athletes agreed to participate in the study.

A structured questionnaire was administered and a physical examination was performed in a reserved room under natural light for the evaluation of the stomatognathic system with the aim of diagnosing injuries stemming from the sport. The athletes were categorised based on level: beginner (white, yellow and orange belts), intermediate (blue belt) and advanced (purple, brown and black belts). The use of a mouthguard was recorded.

The structured questionnaire developed by the authors was used to collect each athlete’s history in the sport (range, participation in competitions, practice time and frequency duration of training), use of mouth guard (in training and competitions, presence of discomfort), history and presence of some type of maxillofacial injury (abrasion on facial skin, contusions, bruises, skin and mucosal lacerations, dental and bone fractures and limited opening of the mouth)^26^. To aid in the diagnosis of centric bruxism (clenching the teeth), physical examination was performed for detection of an increment of the linea alba along the buccal mucosa and teeth marks on the edges of the tongue. Enamel fractures in the cervical region and broken teeth were investigated. The masseter muscle was palpated to determine the occurrence of muscle pain. A functional examination was also performed to obtain the presumptive diagnosis of temporomandibular disorder using an adaptation of the Research Diagnostic Criteria for Temporomandibular Disorders (RDC/TMD), Axis I, which is a validated instrument that enables the identification of patients with a range of simple to complex presentations of TMD. The RDC/TMD edited by Samuel F Dworkin and Linda LeResche, was originally published in 1992^27^. and has since been updated^28^.

The data were analysed using the Statistical Package for the Social Sciences (SPSS for Windows^TM^, version 22.0, SPSS Inc, Chicago, IL, USA). Descriptive and analytical statistics were performed, with the level of significance set to 5% (α < 0.05). Bivariate and multivariate Poisson regression analyses with robust variance were performed to determine associations between the independent variables (gender, age group, athlete’s level, participation in competitions, time of practice, weekly practice, duration of training, possession of a mouthguard, dental arch coverage, use of mouthguard during training sessions and use of mouthguard during competitions) and the dependent variable (occurrence of orofacial injury) following categorisation (α < 0.05). The same statistical approach was used to determine associations between the use of a mouthguard among athletes of Brazilian jiu-jitsu and the independent variables (gender, age group, athlete’s level, participation in competitions, time of practice, weekly practice, duration of training, occurrence of injuries, tooth fracture, traumatic bone injury, laceration of the oral mucosa, skin abrasion on face and limited mouth opening) after categorisation (p < 0.05). The stepwise backward procedure was used in the two regression models, with the incorporation of variables with a p-value < 0.20 in the bivariate analysis. Variables with a p-value < 0.05 in the adjusted analysis were maintained in the final regression model.
